# Is radiography or ultrasonography superior at detecting intestinal obstructions in dogs with acute abdominal signs?

**DOI:** 10.18849/ve.v8i2.483

**Published:** 2023-06-15

**Authors:** Josephine Corrick+

**Keywords:** ULTRASOUND, RADIOGRAPHY, SMALL INTESTINE, OBSTRUCTION, DIAGNOSIS, DOGS, DIAGNOSTIC IMAGING

## Abstract

**PICO question:**

In dogs with acute abdominal signs is radiography or ultrasonography superior at detecting surgical patients with intestinal obstructions?

**Clinical bottom line:**

**Category of research:**

Diagnosis.

**Number and type of study designs reviewed:**

Six relevant studies were identified and reviewed, all diagnostic validity studies. Four had cross sectional designs in place and two have a prospective cohort study design.

**Strength of evidence:**

Moderate.

**Outcomes reported:**

All studies showed that ultrasound and radiography were useful in the diagnosis of small intestinal obstruction in dogs. One study with moderate evidence showed that ultrasound is superior to three-view abdominal radiography for diagnosing small intestinal mechanical obstructions in dogs with acute vomiting (p = 0.013). Most of the studies suggested that ultrasound might be more accurate than radiography at detecting surgical patients with intestinal obstructions, but no sufficient evidence was reported. In some studies, the results are too similar for a statistically significant difference to be claimed without further investigation. All studies suggest that the experience of the person who performs or estimates the diagnostic imaging studies can affect the accuracy of each technique, but no statistical comparisons were made to support this hypothesis.

**Conclusion:**

The results of these studies suggest that both techniques are helpful in the diagnosis of small intestinal obstructions in dogs. There are limitations on each technique and factors that can affect accuracy, like the level of training and expertise but more studies are needed to estimate that. Future studies should focus on the comparison of results when ultrasonography is performed in a general practice setting and knowledge base rather than specialists. The majority of studies included in this summary suggest that ultrasound is generally superior if only one modality can be used, but this is mostly based on weak evidence and further investigations to confirm statistical significance are needed. Considering that all studies were performed by diagnostic imaging experts, the only conclusion that can be safely made is that abdominal ultrasound is superior to three-view abdominal radiographs for diagnosing small intestinal mechanical obstructions in dogs with acute vomiting. Additionally it suggests this modality combined with a good level of training on ultrasonography interpretation or, if possible, cooperation with an expert to get the most out of this tool while treating future patients with relevant issues.

## How to apply this evidence in practice

The application of evidence into practice should take into account multiple factors, not limited to: individual clinical expertise, patient’s circumstances and owners’ values, country, location or clinic where you work, the individual case in front of you, the availability of therapies and resources.

Knowledge Summaries are a resource to help reinforce or inform decision making. They do not override the responsibility or judgement of the practitioner to do what is best for the animal in their care.

## Clinical scenario

A dog presents with acute abdominal signs, due to financial constraints only one diagnostic imaging method can be used; either abdominal radiograph or ultrasonography. Which one is superior at detecting the need for surgical intervention by accurately diagnosing small intestinal mechanical obstruction?

## The evidence

All six identified studies were testing diagnostic validity. Of the six studies appraised the ones with moderately strong evidence are the most recent three (Drost et al., 2016; Elser et al., 2020; and Winter et al., 2017) as they are prospective cohort studies. The other three; Shanaman et al. (2013), Sharma et al. (2010) and Tyrrell & Beck (2006) all had weaker evidence as they all had a cross sectional design with a varied level of blinding involved to try to reduce bias.

## Summary of the evidence

**Elser et al. (2020):** 

Population:	Client owned dogs and cats presenting with clinical suspicion of mechanical obstruction at Matthew J. Ryan Veterinary Hospital of the University of Pennsylvania, USA, between 2008–2015. This paper will focus only on the results of the dog patients.
Sample size:	Dogs: n = 40
Intervention details:	Animals were presenting with abdominal radiographs (ventrodorsal or dorsoventral and left lateral or right lateral views) that were inconclusive for presence or absence of gastrointestinal obstruction. Viewed by radiologist or radiology-resident on duty at the time. Four reviewers were used, two board-certified radiologists with 16 and 10 year’s experience, one board-certified emergency and critical care specialist with 2 year’s experience and one first year radiology resident. Radiographs were subjectively assessed with no objective parameter for obstructions. Reviewers recorded their diagnosis on a Likert scale of 5 categories: Not obstructed; Probably not obstructed; Unsure; Probably obstructed; Obstructed. Reviewers were aware of the study goals but were blinded to all clinical information other than initial concern for obstruction. A second set of abdominal radiographs were taken 7–36 hours after the initial radiographs. The radiographs were reviewed twice: The first phase included reviewing radiographs from both the initial set and the second set of images, one of either set for each patient, to reduce potential for recall bias. The corresponding image for each patient was then reviewed at least a week later. In phase 2, which was at least a month after, the initial and second set radiographs were reviewed again in concert for each patient. Medical management was provided at the discretion of the attending clinician between sets of radiographs. An abdominal ultrasound was done within 3 hours of the second set of radiographs, this was performed by board-certified radiologist or a radiology resident under direct supervision of a board-certified radiologist.
Study design:	Prospective cohort study.
Outcome studied:	Assessment of the initial radiographs was compared to the combined assessment of initial and follow up radiographs in phase 2. The abdominal ultrasound performed in all animals within 3 hours of the second set of radiographs was considered contextualised care. Sensitivity, specificity and percent accuracy of the radiography were calculated for each reviewer at each step; initial radiographic examinations and combined assessment of the two sets of radiographic examinations. Statistical analysis of the area under the curve was done, and agreement between diagnosis measured using Cohen’s Kappa.
Main Findings (relevant to PICO question)	On ultrasound: 11/40 (27.5%) dogs were diagnosed to have a small intestinal mechanical obstruction with ultrasound. One of these dogs was diagnosed with a partial obstruction – this was statistically counted as obstructed. All obstructions were foreign bodies. All of the foreign bodies were non-radiopaque and so were not readily seen on radiographs. The foreign bodies in the dogs included: five linear foreign bodies, one squeaky toy, one rubber material, three non-specified foreign body, one not mentioned. Concurrent foreign material in the stomach, either separate or extending from small intestine was present in 4/11 (36.4%) dogs. On radiographs: The following analysis was made for the entire population of the study which included cats and dogs. Sensitivity for the reviewers on the initial assessment in phase 1 ranged from 36.8–89.5%. Sensitivity for the reviewers for the combined assessment of initial and second set of radiographs ranged from 26.3–84.2%. Specificity for the reviewers diagnoses on the initial assessment ranged from 71.1–94.7%. Specificity for the reviewers diagnoses for the combined assessment ranged from 73.7–97.4%. Accuracy for the initial radiographic examination ranged from 66.7–80.7% across the reviewers. And accuracy for the combined assessment ranged from 70.2–89.5%. Three of the four reviewers increased their percentage accuracy in the second phase of combined assessment as compared to the initial assessment, however this was not statistically significant for any observer. There was poor agreement with one of the reviewers and the other three, and three of the reviewers having moderate agreement between their diagnoses. Presence or absence of an obstruction was not seen to be associated with time interval between the initial and follow-up radiographs.
Limitations:	Population is pre-selected from the radiographic findings where only patients that had a clinical suspicion of obstruction were included that had inconclusive initial radiographs. This would lead to a negative bias towards the use of radiographs in diagnosing small intestinal obstruction, as all cases that were diagnosed on initial radiographs were excluded for this study. Since all patients were treated according to their own individual clinical situation during the interim of sets of radiographs, there was no standardisation of treatment between subjects. Different medical treatment may have had an effect on the second set of radiographs and ultrasound images taken, especially in relation to hydration status. This study looked at the usefulness of taking sequential radiographs in increasing accuracy of diagnoses, one limitation of this study was the length of time between sets of radiographs. There is a wide variety in duration of clinical signs, ranging from less than 24 hours to 3 weeks. This would have an effect on the images taken, worsening of inflammation / thickening of the intestines could have been easier to spot and diagnose with the patients who had a longer duration of clinical signs. While all cases had orthogonal radiographs available for the initial study, there was no standardisation as some were of ventrodorsal and left lateral, some were ventrodorsal and right lateral and one case was dorsoventral and had left lateral. This variety, while more realistic in a general practice setting, does not provide a replicable approach. All of the obstructions were caused by non-radiopaque foreign material, so this study does not include the more obvious cases in which radiograph reviews may be more accurate in diagnosing, reducing its apparent accuracy against ultrasound. The number of patients in this study is low, The number of patients in this study is low, therefore results are not representative of the greater / general population. There was a selection bias of cases that were willing to wait for a second set of radiographs to then be included in the study. This excludes those who may have needed more immediate treatment and included those who may have been more stable. If they had included those more severe cases it may have reflected a more realistic accuracy of diagnosis with radiographs, as they may have contained more obvious causes of obstruction. No universal definition or objective parameter for the diagnosis of obstruction was applied, there were diagnostic limitations for radiographs related to different body conditions, serosal detail and pathology (e.g. free fluid), dogs with inconclusive initial radiographs, patients with foreign bodies and no obstruction could develop obstruction at a later time and show signs only in the follow-up radiographs. The radiologists could not know if a dog just vomited which would change the appearance of imaging, and there is no immediate comparison of ultrasound and radiography at the time of initial presentation. There is also a variety of breeds, ages and numbers of each sex which would not be possible to replicate.

**Winter et al. (2017):** 

Population:	Client owned dogs suspected of having a complete or partial gastrointestinal obstruction without clinically important comorbidities. Presenting at the Small Animal Hospital of the University of Florida, USA between 15 September 2013 and 15 December 2014.
Sample size:	Dogs n = 16.
Intervention details:	Prior to enrolment a history, physical examination, Complete Blood Count (CBC), serum biochemical analysis and abdominal radiography were performed. Dogs were sedated (14 dogs) or anaesthetised (two dogs) prior to ultrasonography and computerized tomography (CT): The dogs had abdominal ultrasound examinations, in dorsal recumbency, performed by a board-certified radiologist or radiology resident under the supervision of a board-certified radiologist. Abdominal CT examinations were done on all dogs, positioned in dorsal recumbency and routine abdominal-volume acquisition protocols were used. All dogs underwent laparoscopy, exploratory laparotomy and surgical treatment of their primary gastrointestinal disease. The ultrasound images were reviewed in real time and findings were discussed with the attending veterinary surgeon. Image analysis and evaluation was performed by a board-certified radiologist. Images were assessed for the presence and location of obstruction and for diagnosis. The ultrasound diagnosis of obstruction was based on subjective assessment of the intestinal dilation, recognition of two distinct groupings of bowel differing in diameter, an identification of intestinal plication or the presence of a foreign body which indicated complete mechanical obstruction only when intestinal dilation of two distinct bowel groupings were also present. CT images were assessed similarly by identifying the presence of intestinal dilation and two distinct groupings of bowel differing in diameter, with or without the identification of a foreign body. Each measurement of bowel diameters and ratios were taken three times and a mean was used.
Study design:	Controlled trial study.
Outcome studied:	Sensitivity, specificity, positive predictive value (PPV), and negative predictive value (NPV) of abdominal CT and ultrasonography for the diagnosis of gastrointestinal obstruction were calculated, with exploratory surgery being considered as reference standard.
Main Findings (relevant to PICO question)	Based on CT and exploratory laparotomy findings 10 dogs had a complete obstruction, three had partial obstruction and three had no obstruction. Sensitivity and specificity of abdominal ultrasound for the diagnosis was 100% and 67% respectively, using exploratory laparotomy as a reference standard. The PPV for ultrasonography was 93% and NPV was 100%. For CT sensitivity and specificity for diagnosis of obstruction was 100% for both, and PPV and NPV were also 100%. There was only one disagreement between modalities, where a false positive diagnosis of an obstruction was made on ultrasonography.
Limitations:	Abdominal radiography was performed prior to enrolment into study, and so standardisation of views and machines used could not be done. Choice of sedation and doses were at the discretion of the attending anaesthesiologist and veterinary surgeon. As the images of the ultrasound exam were reviewed in real time, there may have been some bias due to reviewer not being blinded to signalment and presenting signs of each patient. Having such a small sample size limits the study’s ability to represent a wider population, especially since there were only three dogs without gastrointestinal obstructions included in the study. The patients included in this study are not wholly representative to a wider population as they all presented to a referral hospital instead of a general practice, and so this may not represent the sort of cases and results replicable in a general practice setting. As noted in the study, results of the abdominal ultrasonography can be operator dependent, and an experienced ultrasonographer may not also be available, and so results can differ between reviewers. No statistical analysis on comparisons of accuracy of ultrasonography and CT was performed and so statistical significance cannot be made. There is no comparison between radiology and ultrasound, the nature of the included dogs presented a limitation as all enrolled dogs were suspected of having mechanical gastrointestinal obstruction based on clinical signs, physical examination and abdominal radiography. The study does not mention if previous imaging results were concealed or not, dogs were excluded if they had radiographic evidence of intestinal plication. There is also a variety of breeds, ages and numbers of each sex which would not be possible to replicate.

**Drost et al. (2016):** 

Population:	20 dogs with a diagnosis of mechanical intestinal obstruction. From November 2011 to May 2013: four entire males, five castrated males, one entire female, 10 neutered females. Five dogs were enrolled based on radiographic signs of mechanical intestinal obstruction. Three dogs were enrolled without clinical signs of mechanical obstruction but for which abdominal surgery was planned. The remaining 12 dogs had clinical signs of mechanical intestinal obstruction.
Sample size:	Dogs n = 20. Power and sample size calculator used to get sample size.
Intervention details:	Digital abdominal radiographs and computerized tomography (CT) examinations of the right lateral, left lateral and ventrodorsal views were taken. Surgical exploration of the abdomen was performed within 24 hours of the imaging and the results were used as a reference standard. Three experienced board-certified radiologists reviewed the abdominal radiographs and CT examinations. The reviewers were blinded to clinical history and the images were reviewed at least 12 months after the date images were taken. The images were reviewed in a random order and separately.
Study design:	Case-control study.
Outcome studied:	Abdominal radiographs were evaluated subjectively for signs of gastrointestinal obstructions. With the following criteria: Definitely not obstructed. Likely not obstructed. Equivocal obstruction. Likely obstruction. Definitely obstructed. Should the dog go to surgery: yes or no? Radiographs were then reevaluated using selected objective measurements: Diameter of most distended intestinal segment on lateral projection. Diameter of most distended intestinal segment on ventrodorsal view. Midbody height of L5, width of L5 and width of twelfth rib. After objective measurements, each reviewer was asked again if the dog had a mechanical obstruction with the same criteria above. The same data was also then collected for the CT examinations. Presence of gastrointestinal obstruction was recorded within surgical exploration: Not all dogs had surgical treatment, those that did not were followed for 2 weeks to rule out an incorrect diagnosis i.e. a missed obstruction. Resolution of clinical signs with medical therapy for more than 2 weeks was used as confirmation of a non-obstruction. Surgical results were used as reference standard. Diagnostic sensitivity and specificity for diagnosing mechanical intestinal obstruction using radiographs and CT were calculated for each radiologist and the combined group of radiologists. The subjective criteria above were sectioned into objective criteria: definitely and probably obstructed = obstructed, equivocal, probably not and definitely not = not obstructed.
Main Findings (relevant to PICO question)	17/20 (85%) dogs had abdominal surgery. 8/17 (47%) had small intestinal mechanical obstruction and nine did not. Included in the nine that did not have obstructions were the three dogs scheduled for unrelated abdominal surgery. The 3/20 (15%) dogs that did not have surgery were considered to not have any obstruction after a 2 week follow-up and clinical signs had resolved. So out of the total 20 dogs: eight had small intestinal mechanical obstructions, 12 did not have an obstruction. Sensitivity and specificity for the diagnosis of small intestinal obstruction using CT was 95.8% and 80.6% respectively. Sensitivity and specificity for the diagnosis of small intestinal obstruction using radiographs was 79.2% and 69.4% respectively. Sensitivity and specificity for correctly recommending abdominal surgery after subjective evaluation: Radiography Sensitivity: 91.7%, Specificity: 83.3%. CT Sensitivity: 83.3%, Specificity: 72.2%. Sensitivity and specificity for correctly recommending abdominal surgery after incorporating objective measurements: Radiography Sensitivity: 91.7%, Specificity: 83.3%. CT Sensitivity: 83.3%, Specificity: 80.6%. CT was found to be more sensitive and specific at diagnosing intestinal obstruction, but the difference was not significantly important. For recommending abdominal surgery for obstruction radiography was shown to be more sensitive and specific then using CT but the results were not significantly important. Sensitivity and specificity between radiographic results between reviewers and CT results between reviewers were not significantly important.
Limitations:	Dogs with radiographic signs of a linear foreign body were excluded from this study, which means the findings cannot be representative to all types of mechanical obstructions that present in general practice. Diagnosis was made on a subjective scale, with 3/5 (60%) options stating non-obstructed. This has a bias towards choosing non-obstructed and may have had an effect on the results. The experience of the reviewers is not representative of many clinicians in general practice and so the results have differed with less experienced reviewers. While a power calculation supported the number of animals in the study a larger sample size may have been more representative to a wider population. This study focusses on diagnostic accuracy of radiography and CT and does not include ultrasonography, and so only the accuracy of radiography in the diagnosis of intestinal foreign bodies can be seen without comparison. There is also a variety of breeds, ages and numbers of each sex which would not be possible to replicate.

**Shanaman et al. (2013):** 

Population:	19 client owned dogs. Admitted to Illinois Veterinary Teaching Hospital for acute abdominal signs between December 2010 and January 2012. Aged between 4– 5.2 years old. Eight neutered female dogs. 11 male dogs: four intact, seven neutered. To be involved in the study each animal required the cytologic, survey radiographic and / or sonographic detection of a condition requiring immediate surgical intervention or alternatively sonographic changes consistent with acute pancreatitis or gastrointestinal (GI) neoplasia.
Sample size:	Dogs n = 19.
Intervention details:	All patients underwent abdominal radiography, B-mode and contrast enhanced ultrasonography (CEUS) and multi-detector helical computerised tomography (MDCT). All radiographs as well as computerized tomography (CT) examinations were reviewed in a randomised order by the first author and two board-certified radiologists, all of them being blinded in regards to signalment, presenting clinical signs and final diagnosis. Eight additional digitally acquired three-view abdominal radiographic examinations of normal or non-acute abdominal issues as well as eight CT examinations requiring a minimum of a dual phase protocol, were randomly incorporated for interpretation to prevent reviewer bias but were not included in the statistical analysis. Routine abdominal B-mode ultrasound was performed with a dedicated machine using a microconvex curvilinear array transducer and / or a linear array transducer. The selection of the probe and frequency, depth of field, overall gain, time-gain compensation and focal gain were all adjusted at the choice of the sonographer interpreting at the time to optimise image quality. The ultrasonography was performed and interpreted by either a primary resident or board-certified imaging faculty member at the Illinois Teaching Hospital. Thirdly, the routine B-mode ultrasonography was immediately followed by CEUS. All patients finally underwent MDCT, comprising of a dual-phase contrast enhanced abdominal CT, using a 16-slice helical CT scanner. ‘Every attempt’ was made to scan animals awake – details of which are not described, and intravenous (IV) sedation was used where this was not possible.
Study design:	Cross-sectional study.
Outcome studied:	Variables assessed in radiographic interpretations: presence / absence of pneumoperitoneum, small intestinal plication, visible small intestinal foreign material, presence of small bowel distension, whether it was a surgical or non-surgical case based off image interpretations, peritoneal serosal detail was considered as an ordinal variable: i.e. if normal, focal loss of detail, multifocal loss of detail and generalised loss of detail. Variables assessed by ultrasonography: presence or absence of pneumoperitoneum lesions, hyperechoic mesentery, small intestinal plication, visible zone of transition if bowel plication or distension (only small intestine included), whether it was a surgical or non-surgical case based on interpretations alone, volume of peritoneal free fluid was considered as an ordinal variable (i.e. none, focal, multifocal or generalised). Variables assessed through non-imaging modes: Exploratory laparotomy or necroscopy with histopathology was used as the reference standard for diagnosing surgical underlying conditions such as; small intestine mechanical obstruction, visceral abscess, traumatic diaphragmatic hernia or gastrointestinal perforation. Lesions that were surgically excised and gross pathological lesions were assessed histologically by a single pathologist. Cases that were humanely euthanised had necroscopies to confirm non-surgical conditions. Cases that survived to discharge had overall survival time in days recorded – anything surviving more than 30 days were recorded under the assumption that prolonged survival meant that the underlying condition in the patient did not require immediate surgical intervention. In cases of acute pancreatitis shown by sonographic imaging results of canine Pancreatic Lipase Immunoreactivity (cPLI) and ultrasound (US) guided fine needle aspirate was also required for inclusion into the study. Statistical analysis: Statistical tests and analysis were selected and performed by one of the primary authors. Commercial software was used for statistical analysis and a P-value of < 0.05 was used to be considered statistically significant. Continuous variables were assessed using a Shapiro-Wilk test for normality, and those that that met the assumption for normality a Leven’s test was performed for homogeneity of variance. Categorical and ordinal variable agreement between the modalities was assessed with a Cohen’s Kappa coefficient and weighted Kappa. Interpretation of the Kappa coefficient was split into seven categories: 0 = chance agreement < 0.2 = poor agreement 21–0.40 = fair agreement 41–0.60 = moderate agreement 61–0.80 = good agreement > 0.8 = very good agreement 0 = perfect agreement The agreement between B-mode an CEUS were assessed using a Bland-Altman plot relating to the size of pancreatic lesions. Specificity, sensitivity, positive predictive value (PPV), negative predictive value (NPV) and accuracy for differentiation was also assessed for each modality for if it was a surgical or non-surgical case.
Main Findings (relevant to PICO question)	8/19 (42%) patients of cases were considered to have a non-surgical underlying condition. 11/19 (58%) patients were considered to have a surgical intervention causing underlying condition. 10/11 (90.9%) cases had surgical and / or histologic confirmation of disease. 6/10 (60%) cases were foreign bodies: four linear foreign bodies; cloth, bandage material with sponge, multiple solid objects with fibrous connection, unknown material, one corn cob obstruction in the proximal jejunum, one combined gastric and jejunal cloth obstruction. Radiography results: Survey radiographs correctly identified 8/9 (88.9%) cases as surgical for which a conclusion could be made with imaging findings alone. A single-false negative radiographic diagnosis was made which was a linear foreign body with extension from the pylorus to the distal duodenum. Ultrasound results: Ultrasonography correctly identified 8/9 (88.9%) cases as surgical for which a conclusion could be made with imaging findings alone. The false-negative diagnosis was made in the case of gastric perforation due to an inability to identify pneumoperitoneum. CT results: If a surgical condition could be identified with imaging findings alone, the correct assessment was made by CT in 9/9 (100%) cases. Both radiography and ultrasound were able to identify 8/9 (88.9%) patients correctly that needed surgical intervention. There was moderate to good agreement between modalities with reference against CT as contextualised care, however, both modalities missed one patient for different reasons. Sensitivity, specificity, PPV, NPV and accuracy of each modality for a diagnosis of a surgical condition, excluding two cases for which cytology was required: CE-MDCT = 100% for all. Ultrasonography and radiography together: Sensitivity 89%. Specificity 100%. PPV 100%. NPV 89%. Accuracy 94%.
Limitations:	It is not made clear how 9 cases were found to be surgical, only that imaging was not enough to make a conclusion. Some animals were scanned awake and some sedated, nine animals were imaged awake while 10 received sedation. Evaluation of this new variable may have shown differences in ability to interpret images, and also drug choice may have changed some parameters of the variables interpreted. For example, the use of opioids and possible reduction of gastrointestinal motility. It is not detailed which animals had sedation, or which drugs were used. There is no mention of a power calculation when taking sample size into consideration, even without the calculation it can be seen the sample size is small. The timings between diagnostic imaging are not consistent for all cases and so may have had an impact on what could be seen within the images due to continued gut motility or progression of illness or gross change of the lesions. In 17/19 (89.5%) cases a three-view abdominal series of radiographs were taken (right lateral, left lateral and ventrodorsal views), whereas a two-view series (only right or left lateral and a dorsoventral view) were obtained in the remaining two cases. Having one fewer image to assess may have limited the evaluation for the cases. 18/19 (94.7%) cases had digital radiographs whereas one case had analogue films obtained by the referring veterinarian that were later scanned into the system. This creates an additional variable of image quality and may have had an impact on the results for this case. Radiographs were taken by various people, and so apart from routine procedure guidelines there is not a methodology to replicate, it is also clear by the fact that one case had analogue film sent in that the radiographs were not all taken in the same place and so routine procedure for taking the radiographs could be varied by practice. In 10/19 (53%) cases the evaluation with ultrasound (US) of the abdomen was considered only partial due to inability to visualise specific organs, while not specifically the small intestines this may still have had an impact on visualising surrounding features. Results of ultrasonography and radiographic as individual modalities were not shown in relation to sensitivity, specificity, PPV, NPV or accuracy. Due to the fact that majority of cases required the sonographic detection of a specific lesion to be included in the study there may be bias towards the diagnostic accuracy of ultrasound above the other modalities. Assessment of the 7 grades of how the modalities agree with each other with the Kappa coefficient are subjective. All exams and interpretations were not completed by the same examiner. Ultrasonography interpretation is dependent on the experience and skill of the reviewer and so results in general practice may vary. Duration of clinical signs prior to hospitalisation was variable; and so, timings of imaging from onset of clinical signs are not consistent or replicable. Radiography and ultrasound cannot be performed at the same time and so time between them and movement of patient and possible obstruction could have had an influence on results. In standard practice two orthogonal views are commonly used rather than the three-view abdominal radiography procedure that was performed in the majority in this study. No cases of milder acute abdominal pain were included in the study, there was high heterogeneity of diseases in the study. There is also a variety of breeds, ages and numbers of each sex which would not be possible to replicate.

**Sharma et al. (2010):** 

Population:	Dogs older than 4 months. Admitted to hospital for acute vomiting between February 1, 2008 and May 31, 2009.
Sample size:	Dogs n = 82.
Intervention details:	All 82 dogs had both abdominal radiography and abdominal ultrasound along with one reference procedure: exploratory laparotomy, necroscopy or follow-up phone call to owner at least 1 month after discharge from hospital. Order of imaging was varied due to hospital scheduling. Radiographic and ultrasonographic imaging were performed and interpreted by different blinded board-certified radiologists. Abdominal radiography was performed in left lateral, right lateral and dorsal recumbent positions using one of two radiographic machines and commuted radiography. Positioning order was not standardised. Radiographic examinations were evaluated by board-certified radiologists with a minimum of 10 years experience. Ultrasonography was performed by imaging residents with at least 6 months of training or by a board-certified radiology faculty with at least 6 years of experience. If a resident performed the study, they were supervised by the board-certified specialist. One of two ultrasound machines were used with a variety of transducers. Dogs were placed in dorsal recumbency, fur clipped or parted and coupling gel applied to skin. Practice standardised abdominal ultrasound examination was performed. Laparotomies and necroscopy examinations were performed by attending resident(s) or a board-certified surgeon and pathologist.
Study design:	Cross-sectional study.
Outcome studied:	Variables assessed in radiographic interpretations: Determination of small intestinal mechanical obstruction (subjective measurement). Height of the fifth lumbar vertebral body (L5). Maximal small intestinal diameter. Pattern of small intestinal dilatation. Measurements of the height of the L5 vertebral body (L5) and the small intestinal diameter (SI); And calculation of the ratio of these (SI/L5). Variables assessed by ultrasonography in addition to practice standardised examination: Determination of small intestinal mechanical obstruction (subjective measurement), Potential obstructing lesion (subjective measurement), Number of gastric contractions, Number of small intestinal contractions, Small intestine diameter, Small intestine wall thickness, Small intestine lumen diameter, Small intestine wall layering (subjective measurement), Peritoneal fluid (subjective measurement), Mesenteric echogenicity (subjective measurement). All measurements were objectives except for those noted as subjective. Other outcomes studied: Partial or complete small intestinal mechanical obstruction was confirmed during laparotomy or necroscopy. Dogs were classified as not obstructed if not identified during laparotomy or necroscopy, or if alive and well more than 30 days after discharge from hospital. Statistical analysis was performed using commercially available software. Accuracy of diagnosing small intestinal mechanical obstructions by ultrasound or radiography was determined by calculating area under ROC curve for each modality. Statistical significance was set at P < 0.05 (two sided). Agreement between diagnostic methods was determined used the weighted kappa statistic.
Main Findings (relevant to PICO question)	27/82 (33%) of dogs had small intestinal mechanical obstructions confirmed by surgery (n = 26) or necroscopy (n = 1). Foreign bodies were caused by cloth; plastic bag; toy ball; shoe-string; phytobezoar and dental floss. Three cases were diagnosed incorrectly as small animal intestinal foreign body. There was one false positive based on decisions from ultrasonography alone (found negative in exploratory laparotomy). There was moderate agreement of tests. Radiography produced a definitive result of whether there was an obstruction or not in 58/82 dogs (70.7%). Ultrasonography produced a definitive result of whether there was an obstruction or not in 80/82 dogs (97.6%). With receiver operating characteristic (ROC) curve analysis: Overall accuracy of three view abdominal radiography – area under curve (AUC) 0.82, SE 0.054, 95% confidence interval (CI) 0.72–0.89. Overall accuracy of abdominal ultrasound – AUC 0.95, SE 0.029, CI 0.88–0.99. P = 0.013. Same results between radiography and ultrasound were produced 52/82 (63%). Different results were produced 12/82 (15%). Subjective findings: Confident results (definitely not obstructed, definitely obstructed) were increased after ultrasound exams (n = 80) compared to radiography (n = 58). Intermediate or questionable results (questionably not obstructed, indeterminate, questionably obstructed) were produced more after radiographic interpretations (n = 24) than after ultrasound (n = 2).
Limitations:	Assessment on deciding if there was an obstruction was slightly subjective as the grading was based on confidence of reviewer. All exams and interpretations were not completed by the same examiner. Ultrasonography interpretation is dependent on the experience and skill of the reviewer. While attempts at no conferring between interpreters were made in separating ultrasonography and radiology areas and limiting the available information about patients (interpreters only knew that the patients were vomiting dogs), this study was not completely blinded and bias may have taken place. Duration of clinical signs prior to hospitalisation was variable; and therefore timings of imaging from onset of clinical signs are not consistent or replicable. Sedation and medication inconsistencies were not evaluated or described which may have influenced imaging. This was particularly seen in a dog that was eventually diagnosed with myasthenia gravis. Radiography and ultrasound cannot be performed at the same time and so time between and movement of patient and possible obstruction could have had an influence on results. Positioning was consistent for imaging but not the order of positions, or order of modalities used, this may have also impacted the results. The study included only small-intestinal, not gastrointestinal mechanical obstructions. The foreign bodies might have changed position before or during surgery due to manipulations. There is also a variety of breeds, ages and numbers of each sex which would not be possible to replicate.

**Tyrrell & Beck (2006):** 

Population:	Dogs with clinical signs of a gastrointestinal foreign body, presenting at the University of Melbourne Veterinary Clinic and Hospital (UMVCH) Australia, between June 2003 and September 2004.
Sample size:	Dogs n = 11.
Intervention details:	Dogs presenting to UMVCH that had abdominal radiography and ultrasound with a suspected foreign body were screened for the study. After the foreign body was confirmed, either through removal via endoscope or surgically or it passed naturally in the faeces, the animals were included in the study. Abdominal radiographs were taken for each patient in recumbent lateral and ventrodorsal positions. A complete abdominal ultrasound exam was then performed straight after the radiographs. They were performed by either of the authors using an Acuson Aspen with multifrequency 5–7 MHz microconvex transducer or a linear array 7–13 MHz transducer.
Study design:	Cross-sectional study.
Outcome studied:	Radiographs were analysed by one of the authors (D.T.) for the following changes consistent with a foreign body: Serosal detail, intestinal plication, gastric distension and overdistension of the small intestines. Gastric distension was also recorded if the fundus was greater than three intercostal spaces. Small intestines were recorded as being distended in dogs if the ratio compared to the body of the fifth lumbar vertebrae was greater than 1.6. Accumulation of fine, opaque particulate material was also noted. Ultrasonographic images were assessed for presence and location of any foreign bodies by either author. They were found by identifying their shape, strong distal acoustic shadowing and variable degrees of surface reflection. Gastric and intestinal wall thickness was measured, wall layering was estimated and the presence of gastric and intestinal distension was assessed subjectively. Other assessments were the gastrointestinal contents, lymphadenopathy, intestinal plication, increased echogenicity of the mesentery and if there was effusion present.
Main Findings (relevant to PICO question)	Radiograph findings: 9/16 (56%) cases of a foreign body were detected by radiography, this result includes five cats used in the study. The percentage of dogs found to have foreign bodies through radiography was not defined. Ultrasonographic findings: 11/11 (100%) of the canine cases with foreign bodies were detected with ultrasound. Surgical results: Foreign bodies were removed from the small intestines in nine dogs. One gastric case had the foreign body removed endoscopically, and one colonic foreign body case passed in the faeces. The types of foreign bodies were: Fruit pits, corn cobs, pieces of rubber toy, trichobezoars, an ear plug and elastic meat wrapping. All animals recovered from surgery and clinical signs resolved.
Limitations:	The study did not define the percentage of dogs found to have a foreign body through radiography. There may have been bias when analysing the radiographs as the interpreter was not blinded to the signalment and clinical sign of the patient. While the radiographs were interpreted by one person, the ultrasound examination and interpretation were done by either of the two authors, this may have caused a variation in method and interpretation. This also means some animals could have had both modalities performed by the same person which could introduce more bias into the results. Clinical sign duration varied greatly between the animals, and so timings between onset of signs and imaging are not possible to replicate. There is also a variety of breeds, ages and numbers of each sex which would not be possible to replicate. While the study shows the percentages of correct diagnoses from each modality there are no statistics to show if they are significantly different, and no detail given to which animal or material of foreign body was missed on radiography. Within the ultrasound examination the intestines were considered distended only through a subjective measurement, this could have varied slightly and may have even been biased from other findings in the image, it would be necessary to create a quantitative measurement in order to analyse the degree of fluid accumulation and distension. There were varying types of foreign bodies, while this makes it apply more to general practice it does not focus on how well ultrasound or radiography can detect each one as there is no detail given to which foreign body was missed on radiography, it also makes the study hard to replicate to see if the results are repeated. There is a small sample size and so unlikely to represent a greater population. It was noted that ultrasonic observation of the peristaltic activity may be an important indicator of obstruction, however it was inconsistently recorded in this study and so was not entered as a variable, this may have had an impact on the results if it had been included. Only animals with gastrointestinal foreign bodies were included in the study. This was known to the interpreters. Minimal to no measures were taken to reduce bias from the interpreters. There is also a variety of breeds, ages and numbers of each sex which would not be possible to replicate.

## Appraisal, application and reflection

It is essential to quickly and accurately diagnose a surgical condition such as small intestinal obstructions in dogs presenting with acute abdominal signs, as prompt treatment is necessary in providing the best care and prognosis. It is therefore important to understand which modality would be most accurate in detecting the need for surgical intervention, enabling prompt action in investigating cases further. The most common imaging diagnostic tools readily available in first opinion practice are radiography and ultrasonography. This appraisal summarises the findings from six relevant studies to compare the two modalities for when making those decisions for surgical intervention.

Elser et al. (2020) study population included 40 dogs with clinical suspicion of mechanical obstruction and inconclusive initial radiology, presenting with acute abdominal signs. This study has been included for completeness however, the extent to which it answers the question posed in this summary is limited, this is due to multiple issues including the selection criteria of the patients; patients were included in the study only if there was an inconclusive radiographic finding. This places a bias against the usefulness of radiography to diagnose small intestinal obstruction due to all patients with a conclusive radiograph findings being excluded. Ultrasound was used as the contextualised care and radiograph results were referenced against those findings and accuracy was looked at between interpreters rather than overall. There was shown to be a wide range of agreement among the reviewers, based on their experience level. However, the accuracy of correct diagnosis of intestinal obstruction, measured in a mixed animal population (40 dogs and 17 cats) ranged high from 70.2– 89.7% overall through reviewing radiographs.

Winter et al. (2017) compared ultrasound against computerised tomography CT diagnostic imaging, with the contextualised care of exploratory laparotomy for a definitive diagnosis, this allows the accuracy of using ultrasound to be investigated. Ultrasonography showed to have a sensitivity of 100% and specificity of 67% for a correct diagnosis, whereas CT was 100% for both. This shows ultrasound is an extremely useful tool when investigating dogs with acute abdominal signs when CT is not available.

Drost et al. (2016) compared abdominal radiography and CT, for detection of canine mechanical intestinal obstruction, again using exploratory laparotomy as a reference. The sensitivity and specificity of CT on the diagnosis of obstruction were shown to be slightly lower in this investigation at 95.8% and 80.6% respectively, whereas the radiography sensitivity and specificity were 79.2% and 69.4% but the differences were not statistically significant. If directly compared to Winter et al. (2017) ultrasound results against the same referencing standard ultrasound had a higher sensitivity and a higher specificity for diagnosis. However, both studies investigated CT too and found different results in CT specificity and sensitivity. This shows that there were differences, limitations and possible study design problems between the two. Drost et al. (2016) also had the added evaluation of sensitivity and specificity of recommending abdominal surgery after reviewing the radiograph images, this increased the radiographic interpretation with sensitivity rising to 91.7% and specificity to 72.2%. Therefore, even if a definitive diagnosis cannot be given, the important decision of correct intervention is likely to be chosen. It would have been interesting to compare a similar measurement in Winter et al. (2017) methodology using ultrasound in a similar manner if it had been evaluated.

Shanaman et al., (2013) used a cross-sectional design, this study compared both ultrasonography with radiography against exploratory laparotomy and CT in dogs presenting with intense pain and acute abdominal signs. This study did not only focus on small intestinal obstructions but also a wide range of other underlying causes of acute abdominal signs, but it does address the question of which modality was more accurate by comparing them to the contextualised care of CT. The study showed the level of agreement between the modalities in diagnostic accuracy. Both radiography and ultrasonography correctly identified 8 out of 9 surgical cases, with one false negative for each for different reasons, relative to their limitations. Also, there was shown to be good agreement between the two modalities and CT, which was used as the contextualised care, along with abdominal surgery or necropsy. Finally, the study suggests an impression of superiority from abdominal radiographs compared to ultrasound in cases with pneumoperitoneum, but further studies are needed. This is important as intestinal obstructions that caused intestinal ruptures, which are true surgical emergencies, might be better and more quickly diagnosed with radiographs.

Tyrrell & Beck (2006) used a similar approach by also using a cross-section design to their diagnostic validity study, and this study differs from Shanaman et al. (2013) by focusing purely on gastrointestinal foreign bodies not a wider range of underlying conditions. This study looked at the use of radiography versus ultrasonography to diagnose foreign bodies in small animals, and so included cats and dogs in their population. While the PICO of this Knowledge Summary focuses on dogs it was not defined in their radiographic results what the exact number of positive foreign body findings were in the dog population versus cats. Tyrrell & Beck’s (2006) study give clear results on percentages of the accurate diagnoses of the modalities but it does not have any statistical evaluation of comparing them to each other to see if they are significantly different. Out of the 16 cases, of both cats and dogs, radiography identified 56% of the foreign bodies and ultrasound (US) detected 100% of the cases. All of the cases were confirmed through the removal of the material in surgery, endoscopically, or in one case letting it pass naturally in the faeces. While this study supports the findings found in the other studies that US may be more sensitive in detecting foreign bodies in the small intestine in small animals, it does not evaluate its results through statistical analysis, the differences between the modalities are not proven significantly important, the sample is very small, and the risk of bias is high.

Results found in the study by Sharma et al. (2010) were that 58/82 (70.7%) dogs had a definitive result with radiology showing patients being obstructive or unobstructed in the small intestine. And ultrasonography produced definitive results in 80/82 (97.6%) of dogs. And they concluded ‘abdominal ultrasound was more accurate, with fewer equivocal results and provided greater diagnostic confidence compared with radiography, in small intestinal foreign body mechanical obstruction’.

One limitation that most of the studies have are the small sample sizes, with only one mention of a power calculation in which we can apply the results to a wider population (Drost et al., 2016) and one study with a bigger sample population compared to the rest (Sharma et al., 2010). Both Elser et al. (2020) and Tyrrell & Beck (2006) studies had the inclusion of cats into their sample population as well as dogs, this reduces the sample size relevant to this summaries question even further, and so it may be difficult to apply these findings into a much wider population without future studies supporting these results.

There are a variety of levels of attempts of blinding within each study to consider. Winter et al. (2017) had the ultrasound images reviewed in real time and findings were discussed with the attending surgeon, this may have introduced bias based on signalment and clinical signs as well as introducing additional views and potential influence from other clinicians. However, this bias is reduced with the addition of objective measurements used in the assessment, not just subjective views. In the study by Drost et al. (2016), the reviewers were blinded to clinical history and the images were reviewed at least 12 months after the date of the study and viewed in random order. This strengthens the level of blinding and reduces bias. Shanaman et al. (2013) had various people take the radiographs and then interpreters assessed the images, this creates a level of blinding, where the case details were hidden including the signalment and presenting signs of the animal. The primary author was involved through the three assessed modalities, this could have introduced bias as having looked at the previous modality there could be links made between the previous images unconsciously. So instead there may be confirmation of diagnosis rather an independent diagnosis. However, this hypothesis cannot be proven and gets weaker if we consider that the patient history was unknown and the studies were seen retrospectively. Sharma et al. (2010) had a higher level of blinding in that radiographs and ultrasound exams were not done by the interpreters to reduce bias. With Tyrrell & Beck (2006) it was the authors that performed the exams and interpreted them knowing both the patient history and that all animals included in the study had a gastrointestinal foreign body, so there was no blinding possible to the results of the other modality and of the signalment and presenting signs of the case. The radiographs in the study were all viewed by one interpreter and the ultrasound images were analysed by two different people, this may have had an impact on the results due to method and interpretations.

An important limitation of all the studies is the use of highly trained individuals in reviewing the diagnostic images, in comparison to the wider population in general practice. As seen in Elser et al. (2020) study with the use of four reviewers, with a range of experience from first year radiology resident to board-certified radiologist with 16 years experience, there was found to be a wide range of levels of agreement between the reviewers. And this disagreement was with a group of highly trained individuals, since the first year radiology resident had 6 years of veterinary experience including a 1 year diagnostic imaging specialty internship prior to residency. This shows the degree to which experience has an effect on correct interpretation and decision-making, which is an important limitation when extrapolating the results in this summary into the wider population of general practice. However, there were several more problems and limitations in this study that were previously mentioned, that might have affected its results.

In conclusion, it can be seen with the studies evaluated here that both radiology and ultrasound are highly valuable diagnostic tools that can both increase the accuracy of diagnosis and correct decision-making for surgical intervention. It can also be seen that ultrasound has the potential to be superior than radiography at detecting surgical patients with intestinal obstructions in dogs with acute abdominal signs. This potential is highly correlated to the experience of the observer, and so it is recommended that those in general practice should focus on encouraging development and experience in further imaging training, including ultrasound and radiography assessment for patients with acute abdominal signs in order to increase accuracy and confidence in making future surgical intervention decisions. There are major limitations within the studies observed here; focusing mainly on small sample sizes in most of them, which reduces the ability to apply the findings to larger populations, lack of significant differences between radiographic and ultrasound investigations, or even lack of statistical analysis. While there are differences observed the lack of continuous significant differences highlights the fact that there needs to be further research in this area, ideally with larger sample sizes and without the limitations discussed in this summary.

## Methodology

**Search strategy:** 

Databases searched and dates covered:	CAB Abstracts on CAB Direct (from 1990 – 6 February 2023) PubMed database (from 1990 – 6 February 2023)
Search strategy:	CAB Abstracts: (dog OR canine) (ultraso* AND radiograph*) (obstruct* OR foreign body OR surg* OR acute abdomen) (diagnos* OR detect*) (intestin* OR bowel) 1 and 2 and 3 and 4 and 5 PubMed: (dog OR canine) AND (ultraso* AND radiograph*) AND (obstruct* OR foreign body OR surg* OR acute abdomen) AND (diagnos* OR detect*) AND (intestin* OR bowel)
Dates searches performed:	06 Feb 2023

**Exclusion / inclusion criteria:** 

Exclusion:	Not in English. Pre-1990. Not a direct comparison between ultrasound and radiography to each other or another modality. Not small intestine obstruction. Not including dogs. Not relevant to PICO. Ultrasound as treatment not diagnostic.
Inclusion:	In English. Direct comparison between ultrasound and radiography to each other or another modality. On small intestinal obstructions. Dogs. Relevant to PICO. Diagnostic studies.

**Search outcome:** 

Database	Number of results	Excluded – Not in English	Excluded – No relevant to PICO	Excluded – Not on small intestinal obstructions	Excluded – Not diagnostic comparison study	Excluded – On treatment not diagnosis	Total relevant papers
CAB Abstracts	11	3	1	1	5	0	1
PubMed	65	4	7	21	22	5	6
Total relevant papers when duplicates removed							6

## ORCiD

Josephine Corrick: https://orcid.org/0000-0002-4442-5516

## Conflict of interest

The author declares no conflicts of interest.
